# Assessment of blood lead reference values for the Southern Ghanaian population against the background of international recommendations

**DOI:** 10.1007/s00420-026-02202-w

**Published:** 2026-04-04

**Authors:** Linda Consoir, John Arko-Mensah, Jens Bertram, Julius N. Fobil, Nicole Heussen, Thomas Schettgen, Margot Lakemeyer, Travis Heggie, Thomas Küpper

**Affiliations:** 1 Institute for Occupational, Social and Environmental Medicine, Medical Faculty, RWTH Aachen University, Pauwelsstr. 30, 52074 Aachen, Germany; 2 Department of Biological, Environmental & Occupational Health Sciences, School of Public Health, University of Ghana, P. O. Box LG13, Legon, Ghana; 3 Department of Medical Statistics, RWTH Aachen Technical University, Pauwelsstr. 30, 52074 Aachen, Germany; 4 Center of Biostatistics and Epidemiology, Medical School, Sigmund Freud Private University, Freudplatz 3, 1020 Vienna, Austria; 5 Center for Occupational Medicine, Ecobat Resources Stolberg (ERS), 52224 Stolberg, Germany; 6 School of Human Movement, Sport and Leisure Studies, Bowling Green State University, Bowling Green, OH USA; 7 School of Public Health, Tropical Medicine and Rehabilitation Sciences, James Cook University, Townsville, QLD Australia

**Keywords:** E-waste, E-waste recycling site, Human biomonitoring, Reference values, Heavy metals, Blood lead levels, Ghana, Agbogbloshie

## Abstract

**Background:**

So far there is no reference value for lead (Pb) established for the not occupationally exposed Ghanaian population. This is of special interest and will enable interpretation of data from high-risk areas like the Agbogbloshie e-waste dumpsite. The study suggests preliminary reference values for populations in Southern and Western Ghana.

**Material and methods:**

Blood specimens from non-exposed population were obtained in three key regions (Ashanti-Offinso, Western-Eikwe and Greater Accra-Accra). Three age groups were evaluated (15–24, 25–34, > 34 years). Individual habits (nutrition, occupation etc.) were documented using a standardized questionnaire. Graphite furnace atomic absorption spectrometry was used for Pb analysis.

**Results:**

There was a total of 292 participants (Offinso = 124, Eikwe = 130, Accra = 38). The total mean Pb blood concentration of the Southern Ghanaian population was 43.3 µg/l ± 17.7, with a mean of 48.3 µg/l ± 17.2 for men and a mean of 38.1 µg/l ± 16.8 for women (P = .2.93^–10^). This value is twice as high as the mean Pb level among Germans, and higher than in most European countries. The 95th percentile for the total collective in Ghana was 81 µg/l and 76 µg/l for men and women, respectively.

**Conclusion:**

Based on the 95th percentile of the non-exposed population > 25 years data suggest a preliminary reference value for Pb in Ghana of 81 µg/l for men and of 76 µg/l for women. Since environmental load decreases with increasing distance when unleaded fuel became standard this should be re-evaluated in about 10 years. Future studies should include the eastern and northern regions of Ghana and the analysis of soil for its Pb content as environmental factor.

## Background

It has been estimated that in 2016 just short of 45 million metric tons (Mt) of e-waste were generated worldwide (Baldé et al. 2017). Reliable numbers of how much e-waste is recycled in Agbogbloshie annually are currently still difficult to get. Agbogbloshie is an e-waste recycling site in Accra located in the capital of Ghana. It was rated as one of the ten most polluted places on earth by the Blacksmith Institute in cooperation with the Green Cross Switzerland (Bernhardt and Gysi 2013). As of the report by the E-waste Africa Programme published in 2011, about 215.000 tons of electric and electronic equipment (EEE) were imported into Ghana in 2009. 70% (i.e. 150.500 metric tons) could be reused and sold (Anonymous 2012b). The rest, 64.500 metric tons, ended up as e-waste and was processed in some way or another. Other sources estimate that over 500 containers of e-waste arrive in Agbogbloshie on a monthly basis, of which only a small amount can be repaired and resold directly (Feldt 2017).

Although the total amount of e-waste is unknown in detail it may be accepted that the people living at and around the dump site are significantly exposed to both toxic inorganic and organic chemicals: heavy metals and PCBs etc. through informal recycling. Approximately 50.000 people are living and / or working on the site, which encompasses roughly 16 km^2^ (Feldt 2017). A specific risk factor for public health is the immediate proximity between working and living facilities. In addition to the dismantling and combustion facilities, corrugated iron huts and shelters for living, a school and various markets can also be found on the grounds. Beside a large open food market, there are five large schools in direct neighbourhood of the dump site and thus it may be concluded that heavy metal exposure is also a risk factor for the kids there. This is of special interest since recent data prove that even relatively low blood lead levels (BLL) of less than 50 µg/l are associated with reduced cognitive function (Jeong et al. 2015; Lanphear et al. 2016).

The study is part of the Ghana Environmental Toxicology Project that the RWTH Aachen Technical University has been undertaking together with the Deutsche Gesellschaft für Internationale Zusammenarbeit (GIZ) GmbH and the University of Ghana, Legon, since 2015. The project monitors exposure to heavy metals among people working and living in Agbogbloshie. The project has two main goals:

- To increase awareness of the health risk of heavy metal exposure, especially among e-waste workers and nearby communities.
- To establish regional reference values / baseline for lead (Pb) to estimate the risk of the exposed population since those from other countries (e.g., Germany) cannot be used due to differences in nutritional habits and geological sources. Reference value is defined as a benchmark established through the collection and verification of data from healthy subjects, used to interpret individual test results in a clinical context.

The project was executed in two phases. Phase I examined the exposure to heavy metals of e-waste workers and inhabitants of the Agbogbloshie area. The data of the exposed workers and bystanders at Agbogbloshie have been published elsewhere (Püschel et al. submitted). The authors found increased blood lead concentrations but because of a lack of reference values the interpretation of the results was limited. Phase II focused on the elaboration of reference values for heavy metals in (Southern) Ghana to enable a valid risk estimation for the exposed population. Such a risk estimation is of special importance in Western Africa because several risk factors can be identified here and their combined effect humans may be more deleterious than Pb alone. An example is the combined effect of Pb exposure and malaria-induced anaemia (Ugwuja et al. 2011). Special emphasis was placed on determining the lead concentration in the unexposed population, with the additional aim of answering the question of whether regional differences need to be taken into account due to different dietary habits, economic or geological conditions.

## Material and methods

### Study area and population

In order to establish representative reference values for populations who are not professionally involved in the processing of e-waste or scrap metal, sub-populations from different regions of the middle to southern part of Ghana were established. The selection was based on the following influencing factors:

- Gender (Male/Female)
- Age: 15-24 years, 25-34 years and > 34.
- Residential areas within the country: Offinso, Eikwe and Accra
- Occupational group
- Nutrition

**Fig. 1:**
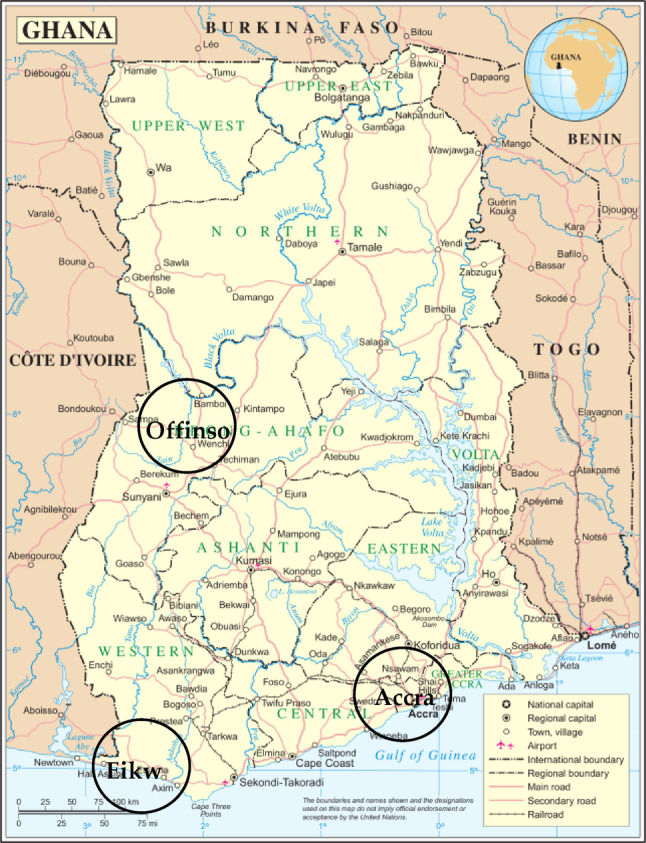
Map of Ghana and the three locations of the study (Department of Peacekeeping Operations Geospatial Information Section (UN) 2005). The map is based on the UN map of Ghana. The locations were amended by the authors of the actual publication

The regions included in this study are illustrated in Fig. 1. The different regions were chosen because different living conditions could potentially lead to different levels of exposure to lead. Offinso is a medium-sized town in a rural setting. Mining, including unofficial gold mining, is virtually non-existent in the surrounding area. The population's diet consists mainly of vegetarian foods (‘fufu’, a firm and starchy porridge made from cassava or yams and plantains) with a small amount of meat (mostly poultry) or fish (dried fish). Eikwe is an extremely rural region with no industry and no mining. The main component of the diet is fresh fish. Accra is a large city with a variety of pollutants to which the population is exposed on a daily basis.

People from the local population were asked via local translators to join. The translators also assisted when the proband’s history was documented and to inform the respective participant about the study to informed consent. Persons with any occupational or private contact to substances which may cause harm to the kidneys were excluded from the study.

### Sample collection and data acquisition

After consenting to the study, blood samples were collected from each participant. Biographical and other data was obtained by means of a uniform questionnaire in standardized interviews. All participants were informed about the purpose and the procedures of the study in detail and signed a declaration of consent. Minors under the age of 18 were only allowed to participate in the study with the consent of at least one legal guardian. It was aimed to get 40 participants per age group and location. Target size was 40 participants at each location. The design was counselled by the Ethical Commissions at RWTH Technical University (EK 093-15) and at Legon University/Accra (MNIMR-IRB CPM 058/14–15).

### Laboratory and statistical analysis

For analysis a graphite furnace ContrAA 700 High Resolution Continuum Source Atomic Absorption Spectrometer (HRCS-AAS) (Analytik Jena, Germany) was used in combination with a standard addition method to determine the levels of Pb in each sample at λ = 283.3060 nm. The limit of quantification (LOQ) and the limit of detection (LOD) were determined by repeated blank measurements: LOD = 3.3 µg/L; LOQ = 10 µg/L.

Data management was performed with Microsoft Access and Excel. The statistical analysis was conducted using Origin Pro8 (OriginLab Corporation, Northampton (MA), USA). After testing for normal distribution subgroup differences were evaluated by using the t-test in case of normal distribution. When data was not normal distributed non-parametric tests (X^2^-Test, Mann–Whitney-U-Test) were used. Divergent distributions were evaluated by applying the Mann–Whitney Test. *P* values < 0.05 were defined as significant.

## Results

### Study collective

**Fig. 2:**
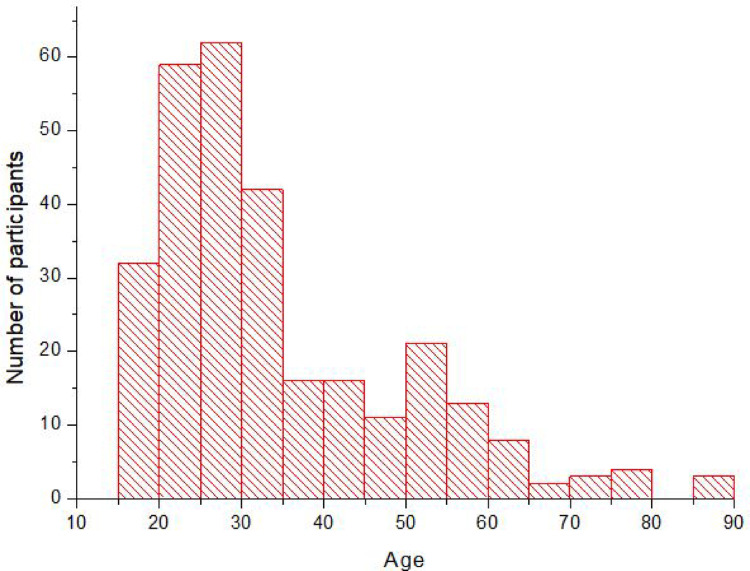
Age distribution of the study participants

The actual field study exceeded the location targets for the Offinso district and Eikwe. It however came short with regards to study participants in Accra due to organizational difficulties and administrative bottlenecks. In total 292 individuals were examined (124 from Offinso, 130 from Eikwe, and 38 from Accra, Fig. 1). With 149 participating men in total, they slightly outnumbered the female participants with 143. The effective number of participants is shown in Table 1. The mean age of the total collective was 33.9 (Sx = 15.0; minimum, median and maximum of 15, 29 and 88, respectively) years of age (Fig. 2). The distributions between the subgroups Eikwe, Offinso, and Accra were not significantly different, i.e., there are no significant differences between the three locations in terms of gender and age nor are there any significant differences of gender and age. The skewed distribution towards the younger ages reflects the demographics of the Ghanaian population in total on one hand (Anonymous 2012a). On the other hand, is it due to the fact that the last age group of the + 34 year olds wasn’t as defined as the other two groups within the study, thus leaving more room for bigger age gaps. Table 2 shows the characteristics of the study collective in terms of lifestyle.

**Table 1:** Total of participants by location, age group and gender

Age group	Gender	Offinso	Eikwe	Accra	Total
15–24	Men	21	22	3	46
	Women	20	20	5	45
25–34	Men	21	24	8	53
	Women	20	21	10	51
> 34	Men	22	21	7	50
	Women	20	22	5	47
Total	Men	64	67	18	149
	Women	60	63	20	143
	All	124	130	38	292

**Fig. 3:**
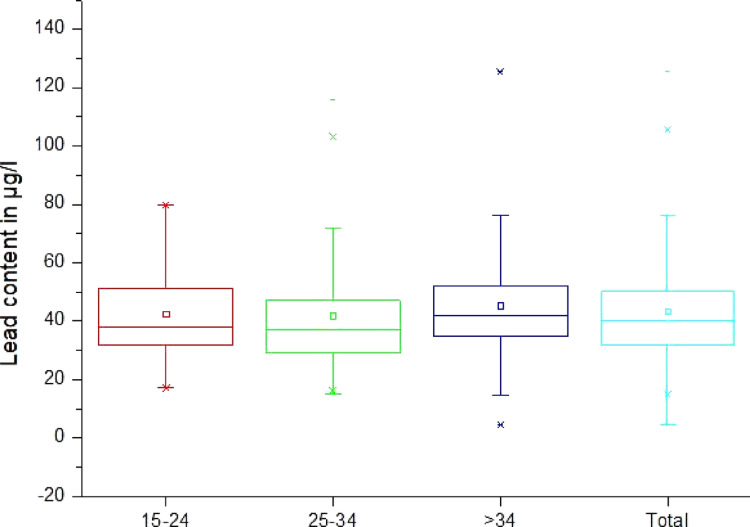
Lead content in µg/l of blood, by age group (Legend of diagram: box range = 25/75 percentile + median; whisker range = outlier; lower/upper line = min/max; lower/upper cross = 1st/99th percentile; square = mean)

**Table 2:** Characteristics of study collective

	Offinso			Eikwe			Accra		
Parameters	15–24	25–34	> 34	15–24	25–34	> 34	15–24	25–34	> 34
*Women*									
Count	20	20	20	20	21	22	5	10	5
Height (geometric mean)	156	161	158	162	161	156	161	163	164
Weight (geometric mean)	61	65	74	69	66	70	71	64	72
Pregnant	0	0	1	13	8	1	0	1	0
Contact with hazardous substances	0	1	2	8	6	12	0	4	4
Engaged in private mining	0	0	0	0	1	0	0	0	0
Accidents/injuries	0	1	6	2	2	1	0	1	2
Numbness, bitter taste	0	0	0	0	0	2	0	0	0
Malaria	6	7	8	12	16	19	5	9	4
Smoking status									
Non-smoking	20	20	20	20	21	22	5	10	5
Smoking	0	0	0	0	0	0	0	0	0
Quit smoking	0	0	0	0	0	0	0	0	0
Fish consumption									
Never	0	0	0	0	0	0	0	0	1
1/month	0	0	0	2	0	1	1	1	0
1/week	1	1	3	0	1	1	0	2	0
> 1/week	19	19	17	18	20	20	4	7	4
Alcohol consumption									
Never	20	18	17	19	20	22	5	9	5
1/month	0	2	0	1	1	0	0	0	0
2–3/month	0	0	2	0	0	0	0	1	0
2–3/week	0	0	1	0	0	0	0	0	0
> 3/week	0	0	0	0	0	0	0	0	0
Stopped drinking alcohol	0	0	0	0	0	0	0	0	0

	Offinso			Eikwe			Accra		
Parameters	15–24	25–34	> 34	15–24	25–34	> 34	15–24	25–34	> 34
*Men*									
Count	21	21	22	22	24	21	3	8	7
Height (geometric mean)	168	171	170	170	168	169	158	171	166
Weight (geometric mean)	56	70	68	60	66	68	69	66	73
Contact with hazardous substances	1	5	4	4	10	12	0	2	2
Engaged in private mining	0	0	0	0	1	0	0	0	0
Accidents/injuries	0	5	2	1	0	0	0	0	0
Numbness, bitter taste	0	0	0	0	0	2	0	0	0
Malaria	14	13	14	16	20	16	3	8	7
Smoking status									
Non-smoking	21	18	20	20	24	21	3	8	7
Smoking	0	1	1	2	0	0	0	0	0
Quit smoking	0	2	1	0	0	0	0	0	0
Fish consumption									
Never	1	0	0	0	1	1	0	0	0
1/month	1	1	0	0	0	0	1	0	0
1/week	2	2	0	1	0	0	0	1	0
> 1/week	17	18	22	21	23	20	2	7	7
Alcohol consumption									
Never	18	10	14	14	15	12	3	5	4
1/month	3	3	4	3	4	1	0	2	2
2–3/month	0	3	0	2	3	3	0	1	0
2–3/week	0	2	3	2	1	5	0	0	0
> 3/week	0	0	0	1	1	0	0	0	0
Stopped drinking alcohol	0	1	1	0	0	0	0	0	1

### Mean lead concentrations of the total collective

The mean Pb concentration was 43.3 µg/l (Sx = 17.71 µg/l; minimum, median and maximum of 4.5 µg/l, 40.3 µg/l and 125.4 µg/l, respectively). The first quartile (Q1) is at 32.0 µg/l whereas the third quartile (Q3) lies at 50.1 µg/l of Pb. The 1st and the 99th percentile correspond to 14.9 µg/l and 105.7 µg/l, respectively.

Table 3 shows the geometric mean of the blood Pb levels (BLL) by location, age and gender. The lowest geometric mean had women over 34 years living in Accra with 33.0 µg/l of Pb in their blood. The highest mean was found in men aged 15–24 in Offinso with 55.6 µg/l. Women had lower concentrations of Pb in their blood than men in most subgroups. Only women aged 25–34 living in Eikwe had a higher mean than their male counterparts with 48.6 µg/l and 47.0 µg/l, respectively (*P* < 0.05).

**Table 3:** Overview of blood lead levels by location, age and gender (geometric mean)

Location/age group	Men	Women	Total
Offinso	50.7	36.4	43.8
15–24	55.6	33.8	45.0
25–34	41.5	34.8	38.3
> 34	54.9	40.7	48.2
Eikwe	48.8	41.2	45.1
15–24	49.5	33.9	42.1
25–34	47.0	48.6	47.7
> 34	50.2	40.8	45.4
Accra	37.6	33.6	35.5
15–24	37.3	33.3	34.8
25–34	37.2	34.0	35.4
> 34	38.2	33.0	36.0
Total	48.3	38.1	43.3

### Lead concentration by age

In a second step the data were categorized by age groups. Age group 3, i.e., the over 34 years olds had the highest mean Pb levels, with mean of 45.4 µg/l, the highest maximum of 125.4 µg/l, but also the lowest minimum with 4.5 µg/l (Table 4, Figure 3). Therefore, this group also shows the highest range with a 120.9 µg/l. The second age group, the 25 to 34 years olds, had a mean of 41.9 µg/l (minimum 14.9 µg/l, maximum 115.9 µg/l, range 101 µg/l). The group with the youngest individuals, group 1, with age range 15 to 24 years, had a mean of 42.7 µg/l (minimum 17.1 µg/l, maximum 79.7 µg/l, range 62.6 µg/l). Table 4 illustrates the numerical data of the different age groups in comparison to the total sample population. At the 0.05 level, the distributions between age groups 15–24 and 25–34 and 15–24 and > 34 are not significantly different (*p*-values = 0.22 and 0.31 respectively), i.e., there are no significant differences between the age groups in terms of BLL. At the 0.05 level, the distributions between age groups 25–34 and > 34 are significantly different (*p* = 0.036), i.e., the over 34 years old have significantly higher BLLs.

**Table 4:** Statistical data of the lead content in µg/l of blood by age group

Age Group	N	Mean	SD	Skew	Min	Q1	Median	Q3	Max	Interq. Range	Range	P1	P95	P99
15–24	9.1	42.7	14.5	0.8	17.1	31.6	38.1	51.2	79.7	19.6	62.6	17.1	72.3	79.7
25–34	104	41.9	19.1	1.6	14.9	29.1	37.1	47.7	115.9	18.5	101.0	16.2	84.9	103.2
> 34	97	45.4	18.9	1.4	4.5	34.8	42.1	52.2	125.4	17.5	120.9	4.5	86.6	125.4
Total	292	43.3	17.7	1.4	4.5	32.0	40.3	50.1	125.4	18.1	120.9	14.9	80.4	105.7

**Fig. 4:**
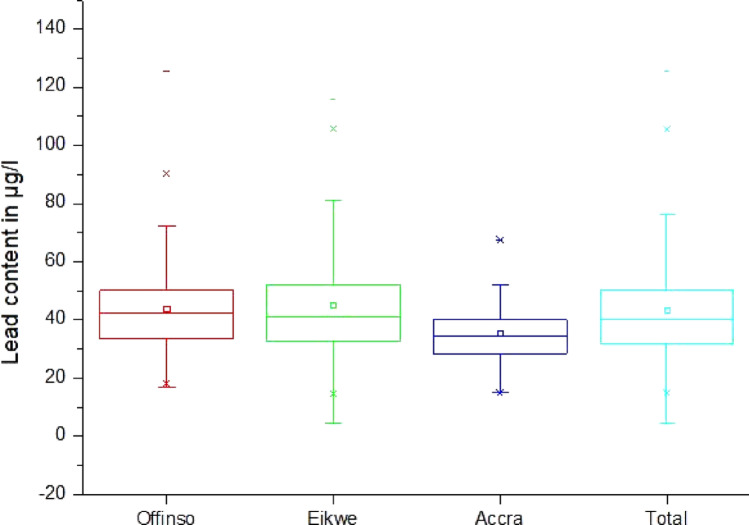
Lead content in µg/l of blood, by location (legend see Fig. 3)

### Segregation of lead concentrations by location

Next, the data were analyzed by location (see Fig. 1 and Table 5). In Offinso the mean BLL was 43.8 µg/l (Sx = 17.0 µg/l, minimum 16.9 µg/l, maximum 125.4 µg/l, median 42.3 µg/l, range 108.5 µg/l). In Eikwe the mean BLL lied at 45.1 µg/l (Sx = 19.5 µg/l, minimum 4.5 µg/l, maximum 115.9 µg/l, median at 41.0 µg/l, range 111.5 µg/l). In contrast, the mean BLL in Accra was 35.5 µg/l only (Sx = 10.3 µg/l, minimum 14.9 µg/l, maximum 67.5 µg/l, median 34.7 µg/l, range 52.5 µg/l). The differences between Offinso and Eikwe were not significantly different (*p* = 0.948) but the differences between Offinso and Accra and Eikwe and Accra were significantly different (*p* = 0.0029 and 0.00235, respectively), i.e., the BLL is significantly lower in the non-exposed population in Accra (Fig. 4).

**Table 5:** Statistical data of the lead content in µg/l of blood by location

Location	N	Mean	SD	Skew	Min	Q1	Median	Q3	Max	Interq. Range	Range	P1	P95	P99
Offinso	124	43.8	17.0	1.4	16.9	33.7	42.3	50.1	125.4	16.4	108.5	18.1	78.6	90.2
Eikwe	130	45.1	19.5	1.3	4.5	32.9	41.0	52.2	115.9	19.7	111.5	14.6	86.6	105.7
Accra	38	35.5	10.3	0.8	14.9	28.3	34.7	40	67.5	11.7	52.5	14.9	52.2	67.4
Total	292	43.3	17.7	1.4	4.5	32.0	40.3	50.1	125.4	18.1	120.9	14.9	80.4	105.7

### Collective by gender

**Fig. 5:**
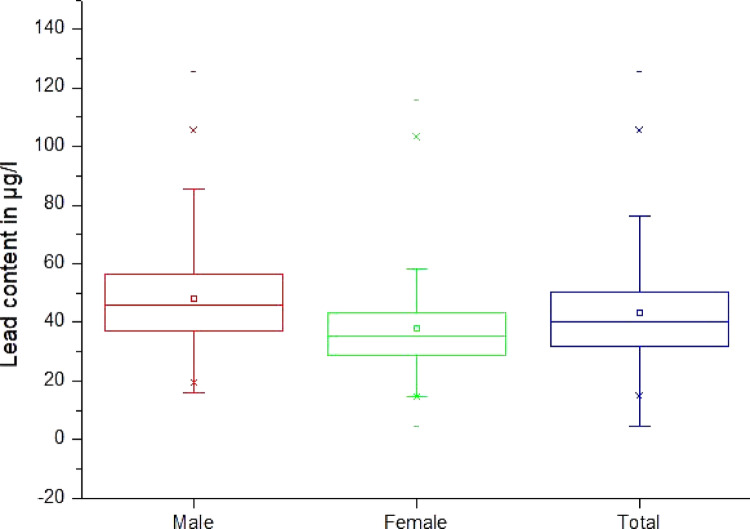
Lead content in µg/l of blood, by gender (legend see Fig. 3)

Men have a mean BLL of 48.3 µg/l (Sx = 17.2 µg/l, median 46.1 µg/l, minimum 16.2 µg/l, maximum 125.4 µg/l, range 109.2 µg/l). Whereas women have a mean BLL of 38.1 µg/l (Sx = 16.8 µg/l, median 35.5 µg/l, minimum 4.5 µg/l, maximum 115.9 µg/l, range 111.5 µg/l (Table 6, Fig. 5). The difference between genders was highly significant (*p* = 2.93^–10^) i.e., men show higher blood Pb levels than women.

**Table 6:** Statistical data of the lead content in µg/l of blood by gender

Gender	N	Mean	SD	Skew	Min	Q1	Median	Q3	Max	Interq. Range	Range	P1	P95	P99
Men	149	48.3	17.2	1.2	16.2	36.9	46.1	56.6	125.4	19.7	109.2	19.5	80.6	105.7
Women	143	38.1	16.8	2.0	4.5	28.7	35.5	43.1	115.9	14.5	111.5	14.6	76.4	103.2
Total	292	43.3	17.7	1.4	4.5	32.0	40.3	50.1	125.4	18.1	120.9	14.9	80.4	105.7

### Collective by smoking habits, fish consumption and malaria status

#### Smoking status

There were only four smokers in the study collective and three ex-smokers. The ex-smokers have the highest mean BLL with 65.2 µg/l followed by the smokers with 55.9 µg/l. Non-smokers have the lowest mean BLL with 42.9 µg/l. The standard deviations are 54.2 µg/l, 30.9 µg/l and 16.8 µg/l respectively. Non-smokers had the lowest minimum of 4.5 µg/l, a maximum of 115.9 µg/l and a range of 111.5 µg/l. Ex-smokers had a minimum of 20.4 µg/l, a maximum of 125.4 and a range of 105.0 µg/l. Smokers have the highest minimum with 26.4 µg/l, the lowest maximum of 85.7 µg/l and therefore the smallest range of 59.4 µg/l. Further details are shown in Table 7. The differences between the various subgroups are not significant. However, taking the small sample size into account this may be an artificial result and should be investigated in future studies.

**Table 7:** Statistical data of the lead content in µg/l of blood by smoking status

Smoking Status	N	Mean	SD	Skew	Min	Q1	Median		Q3	Max	Interq. Range	Range	P1	P95	P99
Non-smokers	285	42.9	16.8	1.3	4.5	32.3	40.2	50		115.93	17.7	111.5	14.9	79.2	103.2
Smokers	4	55.9	30.9	0.0	26.4	29.4	55.8	82.4		85.7	53.1	59.4	26.4	85.7	85.7
Ex-smokers	3	65.2	54.2	1.2	20.4	20.4	49.9	125.4		125.4	105.0	105.0	20.4	125.4	125.4
Total	292	43.3	17.7	1.4	4.5	32.0	40.3	50.1		125.4	18.1	120.9	14.9	80.4	105.7

#### Fish consumption

Most study participants (90.7%) ate fish more than once a week, independent of the location. The highest mean BLL had the subgroup that ate fish once a week (46.7 µg/l). The lowest mean showed the group that only ate fish once a month (39.8 µg/l). The lowest minimum (4.5 µg/l) and the highest maximum (125.4 µg/l) belonged to the group that ate fish more than once a week. They therefore also have the largest range with 120.9 µg/l. The group that ate fish once a month had the highest minimum (29.2 µg/l) and the lowest maximum (78.6 µg/l), thus having the lowest range of 49.4 µg/l. Table 8 summarizes the statistical results. The differences between the groups are not significant, i.e., the BLL seems to be at least relatively independent from the amount of fish in the regular food intake.

**Table 8:** Statistical data of the lead content in µg/l of blood by fish consumptions

Fish con-sumption	N	Mean	SD	Skew	Min	Q1	Median	Q3	Max	Interq. Range	Range	P1	P95	P99
Never	4	43.0	29.0	0.9	19.7	20.4	35.6	65.5	81.0	45.2	61.3	19.6	81.0	81.0
1/month	8	39.8	16.1	2.0	29.2	31.7	34.6	38.9	78.6	7.2	49.4	29.2	78.6	78.6
1/week	15	46.7	18.0	1.0	24.9	32.7	43.1	60.5	88.5	27.8	63.6	24.9	88.5	88.5
> 1/week	265	43.2	17.6	1.5	4.5	32.3	40.4	50.1	125.4	17.8	120.9	14.9	79.7	105.7
Total	292	43.3	17.7	1.4	4.5	32	40.3	50.1	125.4	18.1	120.9	14.9	80.4	105.7

#### Malaria status

The majority of the study participants (67%) had had malaria in the past. This group has a slightly higher mean (43.8 µg/l, Sx = 17.9 µg/l, minimum 14.6 µg/l, maximum 115.9 µg/l, range 101.3 µg/l). In comparison the other group who never suffered from malaria showed a mean BLL of 42.3 µg/l (minimum 4.5 µg/l, maximum 125.4 µg/l, range 120.9 µg/l, see Table 9 for further details). The difference between these subgroups may indicate a tendency but is not significant (*p* = 0.52278).

**Table 9:** Statistical data of the lead content in µg/l of blood by malaria status

Malaria status	N	Mean	SD	Skew	Min	Q1	Median	Q3	Max	Interq. Range	Range	P1	P95	P99
Yes	197	43.8	17.9	1.4	14.6	32.7	40.9	50.3	115.9	17.6	101.3	14.9	85.7	105.7
No	95	42.2	17.3	1.6	4.5	30.15	38.1	49.8	125.4	19.7	120.9	4.5	78.6	125.4
Total	292	43.3	17.7	1.4	4.5	32.01	40.3	50.1	125.4	18.1	120.9	14.9	80.4	105.7

## Discussion

This study aimed to establish reference values for Pb in the blood among non-occupationally exposed Ghanaian populations. This is of interest and critical to survey the general population living in mining regions, but even more importantly, to perform a valid risk estimation for e-waste workers and residents at or near the Agbogbloshie e-waste dumpsite. The collected data among Southern Ghanaian population may be cross referenced with the German population. However, this is a first, and a preliminary approach only, since blood Pb levels are influenced by several environmental and nutritional factors, and these differ significantly between Ghana and Germany. The German reference values for Pb in blood are established by the Umweltbundesamt (Federal Environment Office). They are divided into four main groups: men (40 µg/l), women (30 µg/l), children (girls 3–17 and boys 11–17; 15 µg/l) and children (boys 3–10; 20 µg/l) (Anonymous 2019b). The numbers represent the 95th percentile rounded to the next integer.

The geometric mean of the Southern Ghanaian BLL is 43.3 µg/l for the total collective, a mean of 48.3 µg/l for men and a mean of 38.1 µg/l for women. The 95th percentiles of the Ghanaian population are 81 µg/l and 76 µg/l for men and women respectively. The reference value for children cannot be compared as this study did not include children beneath the age of 15. Considering the 95th percentile, the Southern Ghanaian reference values are thus twice as high in the case of men and 2.5 times as high in the case of women compared to Germany. This difference could have its origin in the different geology (heavy metals in rocks, informal mining…), social status (in Ghana especially when living in the shanty town at Agbogbloshie or in mining areas), drinking water i.e. the source of water supply, the different food intake (particularly fish) as well as the exposure to traffic exhausts (Ahamed et al. 2005). In contrast to Germany, where Pb-free fuel is standard since the 70ties (Hagner 1999), (Lermen et al. 2021) this was introduced in Ghana by May 2004. Therefore, it must be assumed that there is still Pb in the environment of Ghanian regions with intense traffic which environmentally increases the load on the population.

In the United States of America the national geometric mean of the BLL among adults was 8.2 µg/l (95% confidence interval) during the period 2015–2016 (Anonymous 2019a), which was significantly reduced due to decreasing environmental and occupational load of Pb (Kirschner et al. 2013). This relatively low mean is well below the reference value used in the United States of America at that time: In 2015 the National Institute of Occupational Safety and Health, an institution belonging to the Centre for Disease Control and Prevention (CDC), reduced the reference value of the BLL from 100 to 50 µg/l for adults. BLLs above 50 µg/l are defined as elevated (Centers for Disease Control and Prevention 2018). In contrast, according to the Pb standards of the U.S. Occupational Safety and Health Administration (OSHA) workers are to be removed from Pb exposure at much higher values.

It must be differentiated between non-exposed population and persons working with Pb who show concentrations which are increased by several orders of magnitude. Just to give an example: In the U.S. construction industry workers are to be removed at BLLs equal or greater than 500 µg/l, while in general industry this is the case at 600 µg/l. Workers are allowed to return to work when the BLL is below 400 µg/l (Anonymous 2021). Based on their study Kosnett et al. recommend a lower threshold for workers to be removed from Pb exposure with a BLL above 300 µg/l in case of a single measurement and above 200 µg/l if measured in two successive tests over a four-week interval. Furthermore, workers should be removed if exposure control measures over an extended period of time do not decrease the blood concentration below 100 µg/l in order to avoid long-term health risks (Kosnett et al. 2007).

The United Kingdom defines action and suspension levels for general employees (action level = 500 µg/l, suspension level = 600 µg/l) similar to the USA (Anonymous 2020). Lower action and suspension levels are in place for volatile groups such as women of child-bearing age and adolescents and children under 18. In 1995 6868 blood samples of the general public in England were collected for Pb analysis as part of the health survey. The survey showed that the blood Pb levels had fallen substantially since the last survey in 1984. The survey found reference values (95th percentile) of 0.49 µmol/l (i.e. 101.4 µg/l) for men, 0.33 µmol/l (68.3 µg/l) for women, 0.27 µmol/l (55.9 µg/l) for boys and 0.16 µmol/l (33.1 µg/l) for girls (Delves et al. 1996).

Coming back to Pb-free fuel and the long half-life time of Pb in the environment. Even in Europe it is difficult to establish reference values for Pb for a general population. Even after about 40 years since Pb in fuel has been banned the environmental load of the population still decreases and by this any reference value is a provisional one (Rudnai 2019). During this time span the BLL of German young adults dropped from 78.7 to10.4 µg/l corresponding to a decrease of 87% (Lermen et al. 2021). In Sweden several studies were conducted to follow up the concentration of Pb in blood following the phase-out. In repetitive studies over the periods 1978–1994 and 1995–2007 Stromberg observed that the blood Pb level decreased significantly in children aged 7–11. He attributed this decline to the reduction and elimination of Pb in fuel. The last geometric mean of the BLL measured in children was 13.1 µg/l in Trelleborg in 2005 and 13.2 µg/l in Landskrona in 2007 (Stromberg et al. 2008). Wennberg et al. followed up the Pb concentration in blood from 1990 to 2014 in Northern Sweden. For young men and young women aged 25 to 35 the mean BLL was 11 µg/l and 9.69 µg/l respectively in 2014. Men and women aged 50 to 60 had a mean of 15.1 µg/l and 13.1 µg/l respectively in 2014 (Wennberg et al. 2017). A similar tendency must be expected in future for Ghana with the consequence that the reference values suggested in this paper may be adjusted in future.

The influence of nutrients and age has been investigated in Europe, too. France conducted a nutrition and health survey in 2006–2007 with 2029 participants. 80 of those had an occupational exposure to Pb. The “results including occupationally exposed subjects were the same for all percentiles and very close for geometric and arithmetic means” (Falq et al. 2011). For the none-exposed population the total over all adults showed a geometric mean of 25.7 µg/l and a 95th percentile of 73 µg/l. A Spanish nation-wide study conducted from 2007 to 2010 found a “geometric mean of BLLs in the study population of 24.0 µg/l, with women having significantly lower BLLs than men, 19.5 µg/l (95% C.I. 18.5–20.5 μg/l) compared to 28.3 μg/l (95% C.I. 26.7–30.0 μg/l), respectively. Mean BLLs were higher in elder groups in both genders. Women of childbearing age had BLLs of 18.0 μg/l as general mean. Reference values (95% percentile) for Pb in blood in the studied population was 56.8 μg/l, with 64.0 μg/l, 44.8 μg/l and 36.0 μg/l for man, women and women of childbearing age, respectively. Workers from the service sector had lower BLLs than those from construction, agricultural and industry sectors. Small, although significant, geographical differences had been found. In comparison with other European countries, the Spanish population studied had Pb levels similar to populations in countries such as France and Belgium, and slightly lower levels than Italian, Czech, German or UK populations” (Canas et al. 2014).

There are also study groups focusing on human reproduction and (sex) hormones, which analyze the blood level concentration in the general public. A Danish study focusing on women at child-bearing age (18–40 years) found a geometric mean of 8.1 µg/l of Pb in blood and a 95th percentile of 15.8 µg/l (Rosofsky et al. 2017). The sample period included the years 2011 to 2014. A Finish study published in 2016 collected blood samples from 249 adults aged 30–32. The mean blood Pb concentration of men was 17.0 µg/l ± 1.8 µg/l and 9.06 µg/l ± 2.20 μg/l for women (Abass et al. 2017). An international study examined the blood level concentration of 480 women aged 46 to 62 in 2006–2009. The women were living in different cities across Croatia, the Czech Republic, Poland, Slovakia, Slovenia, Sweden, China, Ecuador and Morocco. Between the European cities, the geometric mean varied between 13.5 and 26.9 µg/l. In Guiyang, China, the geometric mean was 68 µg/l, in Camilo Ponce Enriques, Ecuador, the geometric mean was 19.2 µg/l and in Fez, Morocco, the geometric mean was 40 µg/l (Pawlas et al. 2013). The same locations were used by Hubra et al. to examine the BLL in children aged 7–14 (Hruba et al. 2012). Craemer studied the influence of metal exposure on sex hormone levels and the timing of sexual maturation. This one of the most recent studies on BLLs found a geometric mean of 9.3 µg/l and a 95th percentile of 18.7 µg/l in teenagers aged 14 and 15 (De Craemer et al. 2017) (Table 10).

**Table 10:** Geometric means and 95th percentiles of blood lead concentrations across the world

Country		Category	Age	Reference year	N	Geometric mean (µg/l)	95th percentile (µg/l)	References
Armenia, three communities adjacent to metal mining and smelting industries^a^		Children	4–6	2013	159	60.0 S.D. ± 30.0		Grigoryan et al. (2016)
Belgium, Ath (central and peripheral^b^)		Children (c)	2.5–6	2009	49	18.2 95% C.I. 15.9–20.9		Fierens et al. (2016)
		Children (p)			49	14.8 95% C.I. 12.6–17.4		
		Children (c)	7–11		38	15.5 95% C.I. 13.2–18.2		
		Children (p)			36	14.1 95% C.I. 11.8–17.4		
		Men (c)	40–60		27	31.2 95% C.I. 26.4–36.9)		
		Men (p)			25	32.3 95% C.I. 26.1–40.0		
		Women (c)			28	22.5 95% C.I. 18.0–28.2		
		Women (p)			26	20.3 95% C.I. 15.6–26.5		
Belgium, Province of Flemish Brabant		Children	14–15	2012–2015	406	9.26 95% C.I. 8.89–9.64	18.7 95% C.I. 17.4–21.2	De Craemer et al. (2017)
China, Guiyang		Women	46–62	2006–2009	50	68.0 Range: 32.1–230		Pawlas et al. (2013)
Croatia, Koprivnica		Children	7–14	2007–2008	46	17.9 Range: 10–42		Hruba et al. (2012)
		Women	46–62	2006–2009	59	21.4 Range: 8.5–48		Pawlas et al. (2013)
Czech Republic, Prague		Children	7–14	2007–2008	8	15.5 Range: 12–22		Hruba et al. (2012)
		Women	46–62	2006–2009	50	25.3 Range: 10.8–92		Pawlas et al. (2013)
Denmark		Women	18–40	2011–2014	73	8.1	15.8	Rosofsky et al. (2017)
Ecuador, Camilo Ponce Enriques		Children	7–14	2007–2008	69	31.7 Range: 10–130		Hruba et al. (2012)
		Women	46–62	2006–2009	25	19.2 Range: 8.4–42		Pawlas et al. (2013)
Finland		Men	30–32	1997	126	17.0 S.D. ± 1.8		Abass et al. (2017)
		Women			123	9.06 S.D: ± 2.20		
France		Men	18–74	2006–2007	704	30.0 95% C.I. 28.7–31.3	85	Falq et al. (2011)
		Women			1245	22.1 95% C.I. 21.2–23.0	58	
		Men & women	18–39		579	18.7 95% C.I. 17.8–19.6	48	
			40–59		947	29.3 95% C.I. 28.2–30.5	73	
			60–74		423	39.3 95% C.I. 37.7–41.1	102	
		Total	18–74		1949	25.7 95% C.I. 24.9–26.5	73	
		Total (incl. occupational exposure)			2029	25.9 95% C.I. 25.1–26.7	73	
Germany		Children	girls 3–17 & boys 11–17	2014–2017			15	Anonymous (2019b)
		Children	boys 3–10				20	
		Women	18–69	1997–1999			30^c^	
		Men					40^c^	
		Women	18–69	1998		26		Becker et al. (2002)
		Men				36		
		Total			4800	31		
Ghana	Men		15–88	2017	149	48.28 95% C.I. 45.52–51.05 Range: 16.15–125.38 SD: 17.22	80.56	
	Women				143	38.14 95% C.I. 35.39–40.88 Range: 4.47–115.93 SD: 16.75	76.35	
	Total				292	43.31 95% C.I. 41.28–45.34 Range: 4.47–125.38 SD: 17.71	80.44	
Italy	Adults		18–65	2008–2011	1423	19.9 95% C.I. 19.2–20.5	51.7	Bocca et al. (2013)
Morocco, Fez	Children		7–14	2007–2008	39	71.0 Range: 36–200		Hruba et al. (2012)
	Women		46–62	2006–2009	49	40.0 Range: 10.6–130		Pawlas et al. (2013)
Poland, Wroclaw	Children		7–14	2007–2008	27	16.3 Range: 8.0–28		Hruba et al. (2012)
	Women		46–62	2006–2009	51	23.2 Range: 10.0–69		Pawlas et al. (2013)
Slovakia, BanskaBystrica	Children		7–14	2007–2008	57	19.4 Range: 8.0–47		Hruba et al. (2012)
	Women		46–62	2006–2009	52	20.4 Range: 8.9–74		Pawlas et al. (2013)
Slovenia, Ljubljana	Children		7–14	2007–2008	42	13.4 Range: 6.9–24		Hruba et al. (2012)
	Women		46–62	2006–2009	50	26.9 Range: 12.8–110		Pawlas et al. (2013)
Spain	Men		18–65	2007–2010		28.3 95% C.I. 26.7–30	64.0	Canas et al. (2014)
	Women					19.5 95% C.I. 18.5–20.5	44.8	
	Total				1880	24 95% C.I. 23.0–25.1	56.8	
Sweden	Children (Landskrona)		7–14	2007–2008	41	14.0 Range: 6.0–25		Hruba et al. (2012)
	Children (Landskrona)		7–11	2007		13.2 Range: 5.7–58.5		Stromberg et al. (2008)
	Children (Trelleborg)		7–11	2005		13.1 Range: 6.9–29.1		
Sweden (north)	Women		46–62	2006–2009	35	13.5 Range: 5.5–61		Pawlas et al. (2013)
Sweden (north)	Men		25–60	2014	619	25–35 yrs.: 11.0 50–60 yrs.: 15.1		Wennberg et al. (2017)
	Women				926	25–35 yrs.: 9.69 50–60 yrs.: 13.1		
Sweden (south)	Women		46–62	2006–2009	55	18.5 Range: 5.9–49		Pawlas et al. (2013)
UK	Men		≥ 16	1995	3139		101.4	Delves et al. (1996)
	Women				3389		68.3	
	Boys		11–15		180		55.9	
	Girls				160		33.1	
USA	Children		1–5	2015–2016	790	7.58 95% C.I. 6.75–8.5	27.6 95% C.I. 19.4–38.1	Anonymous (2019a)
			6–11		1023	5.71 95% C.I. 5.23–6.23	15.9 95% C.I. 12.4–22.4	
			12–19		565	4.67 95% C.I. 4.33–5.04	11.7 95% C.I. 9.90–13.6	
	Adults		≥ 20		2610	9.20 95% C.I. 8.62–9.82	28.9 95% C.I. 26.5–30.7	
	Men				2488	9.21 95% C.I. 8.64–9.81	29.3 95% C.I. 27.5–32.6	
	Women				2500	7.35 95% C.I. 6.79–7.95	23.9 95% C.I. 22.1–26.5	
	Total		All		4988	8.20 95% C.I. 7.72–8.72	27.5 95% C.I. 25.0–29.8	

^a^Alaverdi, Akhtala and Erebuni district in Yerevan

^b^The study conducted a random sampling in the general population of Ath in two areas: a central area, including two non-ferrous metal plants, and a peripheral area, further away from the plants and presumably less exposed to lead exposure

^c^Based on the environmental survey see (Becker et al. 2002)

Two studies looked at populations affected by Pb exposure. A Belgian study showed that the BLL was significantly higher in young children (age 2.5–6) in the proximity of non-ferrous metal plants than in the periphery. The corresponding Pb concentrations per litre of blood were 18.2 µg/l (95% C.I. 15.9–20.9) and 14.8 µg/l (95% C.I. 12.6–17.4), respectively. No other significant mean differences in metal concentrations were observed between the two areas. The study concluded that “despite higher BLLs in young children living close to the plants, observed metal concentrations remain in the range found in other similar biomonitoring studies in the general population and are below the levels of concern for public health” (Fierens et al. 2016). Another study conducted in 2013 in three Armenian communities adjacent to metal mining and melting industries found a geometric mean of 60 µg of Pb per litre of blood with a standard deviation of ± 30 µg/l. The participants of this study were 159 children aged 4 to 6 (Grigoryan et al. 2016). It can be summarized that levels differ significantly but obviously all of them are well below a concentration which induces harm to the population. This must be taken into account when discussing Ghanaian reference values.

In comparison to most of the European countries listed here Ghana has a higher BLLs in terms of geometric mean and 95th percentile with 43 µg/l and 80 µg/l, respectively. Only the UK data states higher 95th percentiles in all subgroups with 101 µg/l for men, 68 µg/l for women, 56 µg/l for boys and 33 µg/l for girls. However, the data dates back to 1995 and might therefore not give an accurate picture of the current BLLs of the British population which should be lower nowadays. France surpasses the Ghanaian references in two subgroups, namely men with a 95th percentile of 85 µg/l and men and women aged 60 to 74 with a 95th percentile of 102 µg/l. Other French subgroups come close to the Ghanaian reference values. The total French study collective has a 95th percentile of 73 µg/l. The French data was collected in 2007. Not surprisingly, the Armenian children with an environmental exposure to Pb have a higher geometric mean with 60 µg/l than the unexposed population in Ghana. Outside of Europe the middle-aged women living in Guiyang, China, and Fez, Morocco, had a higher geometric mean than the women living in Ghana. The geometric mean of the Chinese women was in fact higher than all Ghanaian subgroups with 68 µg/l. The same counts for children living in Fez, Morocco, with a geometric mean of 70 µg/l. The BLL data from the USA was the most recent one and overall the lowest across all countries.

Within the Southern Ghanaian population it can be said that the age group > 34 has the highest mean and the highest 95th percentile with 45 µg/l and 87 µg/l respectively. This result is not unexpected as Pb accumulates in the bones and blood and tissues are representative for this accumulation. In addition, the > 34 age group has the highest age spread as it is the only group that does not have an upper age limit. The oldest member of this group and the oldest study participant in total is 88, leaving a big life span to get in contact with Pb and to accumulate it.

Measured by the 95th percentile the second group consists of the 25 to 34 years old with a 95th percentile of 85 µg/l and a mean of 42 µg/l. The least amount of Pb content within the blood has the group consisting of 15 to 24 years olds with a 95th percentile of 72 µg/l and a mean of 43 µg/l.

Location wise, the group from Eikwe has the highest Pb content in the blood with a 95th percentile of 87 µg/l and a mean of 45 µg/l. This could be due to a higher percentage of fish in nutrition. However, this assumption has not been backed up by the data. In addition, the information gathered about the fish intake has not been accurate and differs widely even at the same location and additionally Pb content of fish varies by region. Since the Pb concentration of the fish in the respective region have not been measured this cannot be excluded as variable source of Pb. However, data indicate a significant Pb concentration in fish in Ghana and the Gulf of Bengal. The risk for the population depends on the amount of fish in the food, the fish species, and the location where the fish was living (Nyarko et al. 2023; Steinhausen et al. 2022; Kortei et al. 2020; Gbogbo et al. 2018; Okyere et al. 2015). This is also supported by data from rural regions in Southern Brazil (de Almeida Lopes et al. 2015). Although Pb intake by fish varies significantly this potential exposure should not be ignored.

Other reasons for the high Pb content could be smoking. The actual data cannot establish a correlation between smoking habits and Pb content in the blood. Out of a total of 292 study participants 285 were non-smokers, four were active smokers and three had quit smoking. This portion of smokers is a bit less than one would expect since data show about 8.5% smokers in the African population in 2020 (https://de.statista.com/statistik/daten/studie/716370/umfrage/raucheranteil-nach-who-region-weltweit/). Global State of Tobacco Harm Reduction (GSTHR), an independent, U.S. nonprofit 501(c) (3) grant making organization states that there has been a downwards trend in current smoking prevalence in the general population in Ghana (https://gsthr.org/countries/profile/gha/, last update Dec 11th, 2025). In 2000 the prevalence was estimated to be 5%; this decreased to 3.6% in 2015, with a projected decrease to 3% by 2025. Men's smoking prevalence decreased from 10 to 7% between 2000 and 2015, and is projected to decrease further to 6% by 2025 according to WHO trend data. Women's smoking during the same period remained low at just under 1% in 2000, and is projected to decrease to 0.1% by 2025.

In the current study, women and men were represented in roughly equal proportions. Assuming a prevalence of 3.6% smokers in the total population according to GSTHR at the time of the study, 10–11 smokers would be expected among the 292 people examined. There is no clear reason why only 7 smokers were recorded in the collective but with such small numbers, the most likely explanation is the random selection of participants. At the very least, the collective reflects the situation in the country very well. However, with such a small subcollective of 7 smokers in the actual study it was impossible to prove a correlation and therefore this must be open for future research. However, from other studies it is known that levels of Pb, cadmium, and mercury are increased in smokers (Almerud et al. 2021).

The subgroup with the lowest BLL is the group from Accra. This group has a 95th percentile of 52 µg/l and a mean of 36 µg/l. With only 38 study participants this location has the fewest number of results and therefore data must be interpreted with care. Furthermore, the distribution amongst the age groups is not even. 68.4% of the participants belong to the two younger age groups, i.e. groups 15 to 24 and 25 to 34 years. Only 31.6% belong to the oldest age group > 34. The lack of lifetime in Accra in comparison to the other locations could be one of the reasons for the lower Pb content in the blood. Another reason for the lower Pb content in the blood could be a higher level of education and social status with easier and regular access to clean drinking water. Such collective typically has a potentially coherent awareness of environmental and biological damage caused by an over exposure to hazardous substances. The younger collective of Accra was also born short before Pb was eliminated from fuel and therefore this exposure is significantly lower.

With regards to the sex of the participants in our study, men had a higher Pb content in the blood with a 95th percentile of 81 µg/l and a mean of 48 µg/l than women with a 95th percentile of 76 µg/l and a mean of 38 µg/l. All female study participants were non-smokers. The two oldest study participants were women aged 88 and 86 with a BLL of 37 µg/l and 43 µg/l, respectively, thus not showing elevated BLLs. Out of the ten oldest study participants 60% were women with a mean BLL of 30 µg/l. The four remaining men had a mean BLL of 84 µg/l. One reason might be their occupational exposure, another one their red cell count. However, even though the occupations were captured by gender and some occupations were primarily executed by women, such as the field of midwifery/nursing (53 women versus 23 men engaged in this field of work), it cannot be deducted from the data that women generally are involved in less Pb exposed fields of work. For example, a relatively large number of young men (n = 23) were still enrolled in senior high school and another big occupational group was comprised of men working as security guards (n = 11). The study remains short of explanations how the different occupational groups are tied into exposure to Pb.

Based on the data described above a blood lead concentration of 81 µg/l for men > 25 years and of 76 µg/l for women > 25 years is suggested as reference value (95% percentile of the adult population included) for the general population in Ghana, i.e. people without additional exposure for example by occupation. As stated above the reference value is defined as a benchmark established through the collection and verification of data from healthy subjects, used to interpret individual test results in a clinical context. The aim of the current study was to facilitate the interpretation of clinical epidemiological data by establishing a reference value for the unburdened normal population, as it must be assumed that significant exceedances of this limit established for the healthy population may lead to health impairments. A valid risk assessment whether quantitatively or at least as a trend is not possible based on the available data. This can only be carried out once clinical data is available that correlates blood lead concentrations in the population with lead-associated symptoms and findings. Until clinical-pathological data correlating with the BBL is available, this reference value should be used as a limit that the inhabitants of Ghana should not exceed. If corresponding studies show that lead-related damage is also diagnosed at this limit value, there must be a discussion at the political level on how to reduce lead exposure, which is currently unavoidable due to environmental factors. In some cases, this will happen automatically as a consequence of the phasing out of leaded fuels over the years.

## Limitations of the study

Originally, the study intended to establish the reference values for the whole of Ghana, comprising the West, the North, the South, the East and the capital of Accra. Due to long distances and partially difficult roads as well as weather conditions such as rainy season due to the African monsoon the project had to be limited to the South and West of Ghana. The two additional regions looked at to complete the reference values for the Ghanaian population are Tamale in the North and Volta in the East. Both regions demonstrate further unique geographic, cultural and geological particularities: the North with its dry landscape of rocks and sand and the Volta region as a wet, swampy area with the Volta River and its delta. Therefore, these regions should be included in future studies.

In the actual study the investigation of the local soil was not included. Since this may be an important environmental source of heavy metals at least some probes at different locations should be included in future projects.

Within the project, Accra has significantly fewer study participants than the other two locations. This is due to time and organizational constraints the expedition group was facing whilst travelling. Here also additional data should be obtained which also should fit better for social factors with the groups investigated in the actual study.

With regards to the questionnaire, it was challenging to communicate with a substantial part of the rural population. Depending on whether a tongue is counted as dialect or language there are 44 to more than 100 languages spoken in the country and no lingua franca which is spoken by everybody. There have been situations in which two translators were necessary: One person communicating with the study participant in his/her dialect and then translating the answer into another dialect to a third person, who was then able to translate the answer into English. The answers were noted down according to the interviewer’s best knowledge and understanding but this procedure may have caused a bias, especially for more “critical” questions like habits of nutrition. This is especially true for the quantity of fish being consumed. The answer might not have been realistic and there may be a significant recall bias.

There are several factors which may cause increased serum creatinine levels and some data indicate that it may be unchanged even in persons with high lead exposure but with less than 50% decrease of glomerular filtration rate (Pergunde et al. 1993). Another problem is that so far a lead-induced nephropathy has not yet been defined by diagnostic criteria (Pergunde et al. 1993; Nuyts et al. 1991). Therefore some authors suggest to add tubular markers like α1-microglobulin, N-acetyl-β-D-glucosaminidase (NAG) ribonuclease and/or Tamm-Horsfall glycoprotein which are increased in 30% of chrnic lead exposed persons (Pergunde et al. 1993).

## Conclusion

A blood lead concentration of 81 µg/l for men > 25 years and of 76 µg/l for women > 25 years is suggested as reference value (95% percentile of the adult population included) for the general population in Ghana, i.e. people without additional exposure for example by occupation.

Due to a further decreasing environmental load with more and more time since Pb-free fuel became standard this should be re-evaluated in about 10 years. Further studies should also include the northern and eastern regions of the country.

## Data Availability

Data are available on request.
